# Effect of CO_2_ Laser Treatment on the Fabric Hand of Cotton and Cotton/Polyester Blended Fabric

**DOI:** 10.3390/polym9110609

**Published:** 2017-11-13

**Authors:** On-na Hung, Chi-wai Kan

**Affiliations:** Institute of Textiles and Clothing, The Hong Kong Polytechnic University, Hung Hom, Kowloon, Hong Kong, China; anna_hung819@hotmail.com

**Keywords:** fabric hand, PhabrOmeter, CO_2_ laser, cotton, polyester, woven, blended fabric

## Abstract

This paper compares the impact of laser treatment on cotton and cotton/polyester blended fabric hand properties, using the PhabrOmeter system. Five fabric hand properties, namely, stiffness, smoothness, softness, wrinkle recovery rate, and drapability, were obtained, and it was proven that laser treatment could be successfully used to change the fabric hand. In the case of pure cotton woven fabrics, the fabrics were found to have better drapability and wrinkle recovery after laser treatment. In cotton/polyester blended fabrics, stiffness was found to be relatively higher after laser irradiation.

## 1. Introduction

The aim of this study was to evaluate the impact of laser treatment on fabric hand properties of plain pure cotton and cotton/polyester blended fabrics. Carbon dioxide (CO_2_) laser was used with different intensities in terms of resolution and treatment time. Due to the increasing concerns about pollution and protection of the environment, the need for environmentally friendly methods for surface treatments is established. Laser treatment, based on physical principles, offers advantages over conventional chemical methods. It enables precise surface modification in a short time. It is easy to apply and control and is totally environmentally clean with no consumption of water or chemicals [1,2,3,4,5].

Fabric hand has been recognized as one of the most important performance attributes of apparel textiles. It is defined as “perceived overall fabric aesthetic quality” [6]. The quality of the textile material is mainly perceived by touching and has a direct impact on the product’s appeal. Fabric hand not only affects the perception of consumers towards the products, but also development from the stage of design to manufacturing and merchandising through to the final consumer.

Research works on fabric hand have used mainly two measurement approaches: subjective and objective assessments. Fabric hand obtained by subjective assessment is a traditional procedure in which trained handle experts describe the fabric quality. However, according to Pan [7], fabric hand is defined as the human tactile sensory response towards fabric, which involves not only physical but also physiological, perceptional, and social factors, which largely influence the accuracy and repetitive nature of the result. Since the subjective assessment is easily influenced by many factors, objective assessment methods like the Kawabata Evaluation System for Fabric (KES-F), the Fabric Assurance by Simple Testing (FAST), and PhabrOmeter systems have been developed. These systems quantify the fabric hand in terms of low-stress mechanical properties, including bending, shear, tensile, and surface properties [8,9,10]. Unlike the complex and lengthy KES-F and FAST measurement processes, the newly developed PhabrOmeter system has overcome these limitations as it can rapidly evaluate fabric hand properties [11]. A PhabrOmeter instrument is a fabric sensory quality evaluation system developed by Nu Cybertek, Inc. in Davis, CA, USA, and has become a designated machine by American Association of Textile Chemists and Colorist (AATCC) standard for fabric hand evaluation [12]. The PhabrOmeter system measures the “feels and looks”, or so called “sensory perceptions”, of such sheet type fibrous products. The PhabrOmeter system consists of an instrument and a software package. The system is based on the pattern recognition theory, by extracting the quality characteristics of the product, and connecting them to the human sense, so as to provide fast and reliable quality evaluation results. It fills a very important blank [12].

This system can measure both woven and knitted fabrics, and is capable of testing fabric hand objectively and obtaining the data in terms of five handle features, namely, stiffness (a higher value of stiffness refers to a stiffer fabric), smoothness (the larger the smoothness value, the smoother the fabric is), softness (the smaller the softness value, the softer the fabric is), drape (the smaller the drape value, the better the draping or shape of the fabric will be), and wrinkle recovery rate (a larger value of wrinkle recovery rate value implies higher resistance to wrinkling of the fabric) in one-time testing [13].

In order to study the effect of laser treatment on fabric hand, the PhabrOmeter fabric evaluation system is used for measurements in this study. By understanding the alteration of different properties of fabrics through laser treatment, better choice can be made between pre- and post-laser treatment in order to obtain the most desired fabric hand effect.

## 2. Experimental

### 2.1. Materials

Two types of ready-for-dyeing woven fabrics were purchased from Lai Tak Enterprises Limited, Hong Kong, China. The fabric specifications are as shown in Table 1.

### 2.2. Sample Preparation

The fabric samples were soaked with 30 mL/L acetone (Sigma-Aldrich, Hong Kong, China) for 10 min to remove any grease and dirt on the surface. After washing, the samples were rinsed with water and hydro-extracted in a Nyborg C290R Hydro-extractor for 5 min. Lastly, the samples were dried in a Nyborg T4350 tumble dryer (Electrolux, Stockholm, Sweden) for 15 min. All cleaned samples were conditioned under standard conditions of 65 ± 2% relative humidity and 21 ± 1 °C temperature for at least 24 h prior to all experimental and evaluation tests.

### 2.3. Laser Irradiation

A commercial pulsed CO_2_ source laser engraving machine (GFK Marcatex FLEXI-150, Jeanologia S.L., Valencia, Spain) was used under atmospheric conditions, coupled to an Easymark 2009 laser system (Rofin, Hamburg, Germany). The fabric samples were treated at different combinations of processing variables (resolution and pixel time), resolutions 52, 60, and 68 (dpi) and pixel time of 110, 120, 130, and 140 (µs), i.e., 12 combinations in total.

### 2.4. Fabric Thickness

Fabric thickness was measured by a thickness tester (Hans Baer AG, CH-Zurich telex 57767, Zurich, Switzerland) according to the ASTM D1777-96. Three specimens were used and the thickness of each specimen was measured at 10 different positions. Finally, the averaged thickness of each specimen was calculated. The change of fabric thickness was calculated based on Equation (1):

(1) $$\mathrm{Thickness}\;\mathrm{change}\;\left(\%\right)=\frac{T-T_{o}}{T_{o}}\times 100\%$$

where *T* (mm) refers to the thickness of the sample after treatment and *T*_o_ (mm) refers to the original thickness of the sample.

### 2.5. Fabric Hand Measurement

Fabric hand properties were measured by the PhabrOmeter Model 3 fabric evaluation system (Nu Cybertek Inc., Davis, CA, USA) with software 3.7.1 according to the standard AATCC test method 202-2012. Three circular specimens with a diameter of 113 ± 2 mm were randomly cut from each fabric sample followed by testing, and were then averaged. Quantified data including stiffness, smoothness, softness, wrinkle recovery rate, and drape index were obtained with the following definitions.

Stiffness—Any material that is easily bent may be described as flexible, limp, or pliable.

Smoothness—Surface friction is resistance of surface to slipping. It can be thought of as how hard you have to push your fingertip to move it across a fabric.

Softness—Compressibility may be judged by squeezing a crumpled piece of fabric in your hand.

Wrinkle Recovery Rate—After the first measurement of a given fabric sample, the sample is allowed to recover for 5 min (ASTM) and is tested again.

Drape Index—The extraction test is in fact a forced drape, and so it should be able to describe the fabric dynamic drape behavior. The test results can be used for drape test and comparison.

### 2.6. Scanning Electron Microscopy (SEM)

Surface morphology of the fabric was investigated by SEM (Lecia Stereoscan 440, Cambridge Instruments, Cambridge, UK) with a resolution of 3 nm at 40 kV.

## 3. Results and Discussion

### 3.1. Cotton Fabric

#### 3.1.1. Stiffness

**Figure 1:**
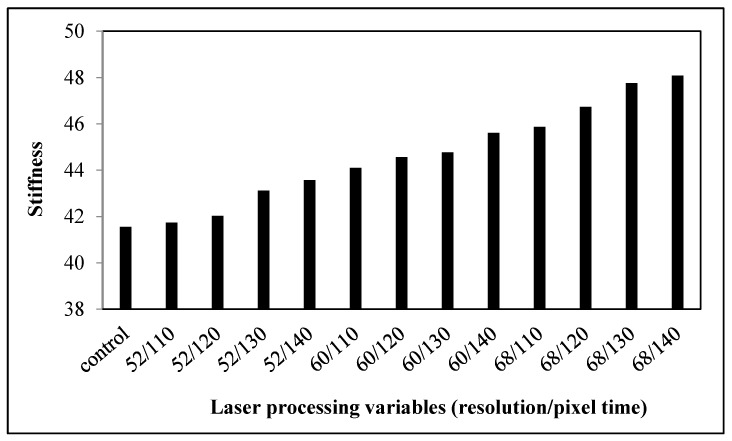
Relationship between the laser processing variables and stiffness of cotton fabric.

The stiffness of the control and the laser-treated fabrics is presented in Figure 1. According to the PhabrOmeter system, the higher the value of stiffness, the stiffer the sample will be. Stiffness refers to the resistance offered by a material to a force tending to bend it [14]. Bending of the fabrics is affected by the mobility of warp and weft yarn, which is influenced by the fiber-fiber friction. With a greater fiber-fiber friction, the ability of the fibers to slide past each other during yarn and fabric deformation diminishes. This leads to a better resistance to deformation and consequently greater stiffness.

**Figure 2:**
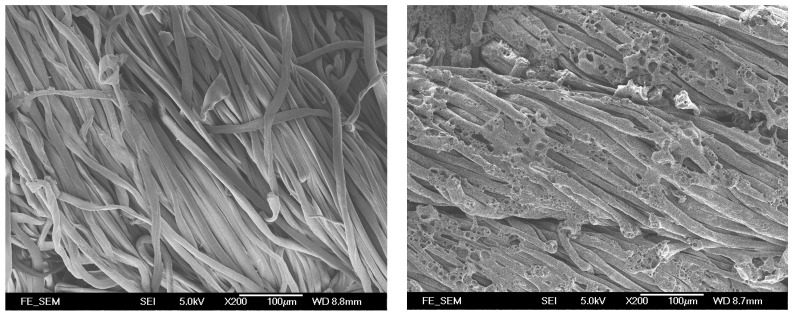
Morphological feature (200×) of control cotton fabric (**left**) and laser-treated cotton fabric with variables of 52 dpi/120 µs (**right**).

The control fabric had stiffness value of 41.55. The control fabric had a lower stiffness value than most of the laser-treated fabrics, which demonstrates that the fabrics become stiffer after laser treatment [11]. Rigidity is affected by the friction coefficient between fibers forming the yarns and yarn diameter [15]. The higher rigidity is due to the etching effect caused by the laser treatment, as different sizes of pores are formed on the fiber surface leading to higher surface friction [16,17,18]. This is proven by the SEM image of laser-treated cotton shown in Figure 2. The increase in the coefficient of friction between fibers results in the lower mobility of yarns. The increase in stiffness after laser treatment means the fabric becomes more difficult to sew and less comfortable to wear.

As far as the laser processing variables are concerned, stiffness values of laser-treated fabrics are enhanced gradually when the resolution and pixel time are increased. It is also proved from the SEM images that when the resolution was increased and pixel time was prolonged, the density and sizes of pores formed on the fiber surface were enhanced [18]. The reason for this is that with the increase in resolution (dpi), more laser spots are produced in one inch. This leads to continuous dehydration and oxidization, resulting in the formation of more pores [18]. This consequently increases the surface friction and thus the stiffness value [10]. Among all the laser-treated samples, the sample treated at 68 dpi/140 µs has the highest stiffness value, which means that this fabric is the stiffest.

#### 3.1.2. Smoothness

**Figure 3:**
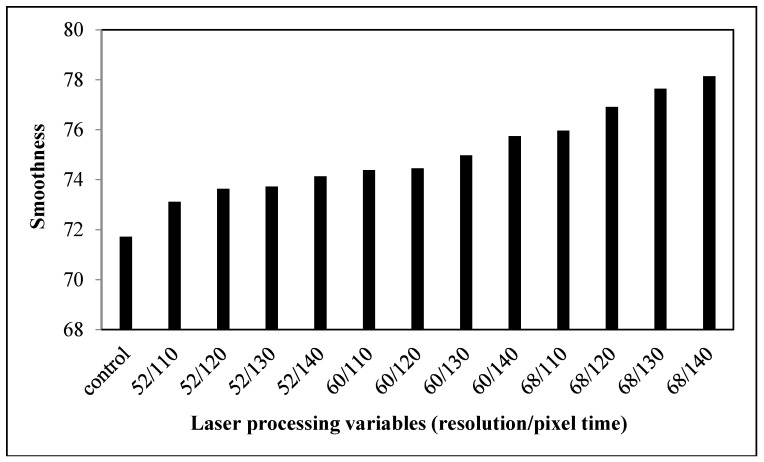
Relationship between laser processing variables and the smoothness of cotton fabric.

Smoothness describes surface friction or resistance to slipping. The larger the smoothness value, the smoother the fabric is. Figure 3 shows the smoothness value of the control and laser-treated fabrics. The control fabric has the lowest smoothness value but, after laser treatment, the smoothness value increases. This indicates that laser treatment enhances the smoothness of the cotton fabric surface. As laser treatment has an etching effect on fabric surfaces, all fibers protruding from the fabric surface are etched away (Figure 2) [18]. With the increment of resolution (dpi), more laser spots are produced in one inch which would result in dehydration and oxidization for removing surface protruding fibers [18]. Therefore, the smoothness of fabric surface was enhanced, making the surface of the fabric more even.

When the effect of laser processing variables is considered, resolutions of 52 dpi, 60 dpi, and 68 dpi show similar effects when the pixel time is altered from 110 µs to 140 µs. The smoothness is thus enhanced. As a result, the increase of laser processing variables within the range of 52 dpi/110 µs to 68 dpi/140 µs improves the smoothness of cotton fabric.

#### 3.1.3. Softness

**Figure 4:**
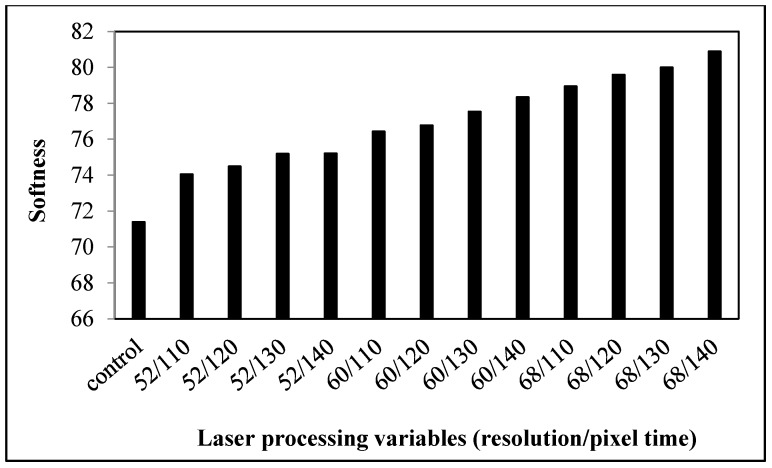
Relationship between laser processing variables and the softness of cotton fabric.

Softness is the flexural property of the fabric and is one of the factors related to the determination of the wear comfort of clothing. It is defined as compressibility, i.e., the smaller the value, the softer the fabric will be. All laser-treated fabrics have a larger value than the control fabrics (Figure 4), implying that the fabrics have a slightly harsher surface after laser treatment, which reduces the softness during physical touching [13]. This decrease in softness may be due to the etching effect of fibers caused by laser treatment. The softness value of fabrics is found to have further increased when the laser processing variables are enhanced, implying that the softness of fabrics decreases. Since thickness is one of the factors affecting the softness, laser treatment with high energy causes a larger reduction in thickness (the thickness may change from 0.52 mm to 0.44–0.47 mm after laser treatment) [10] and hence lowers the softness of the fabric [17].

#### 3.1.4. Wrinkle Recovery Rate

Creasing/wrinkles of a fabric during wear do not constitute a desirable change in appearance. Cellulosic materials like cotton have poor resistance to creasing.

**Figure 5:**
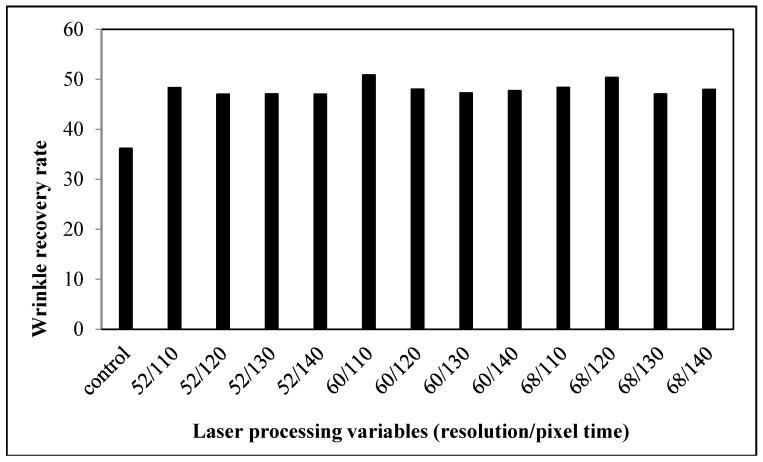
Relationship between laser processing variables and the wrinkle recovery rate of cotton fabric.

The wrinkle recovery value measures the wrinkle resistance of the fabric. A larger wrinkle recovery rate implies higher resistance to the wrinkling of the fabric. This value is determined by measuring the fabric hand and drape of the fabric twice with a given time interval between measurements. During the test, the fabric is subjected to a pulling force and experiences complex wrinkles. After it is given a recovery time, the differences in terms of the defined fabric hand variables are measured again when it is tested for the second time. As shown in Figure 5, the control cotton fabric has a lower wrinkle recovery value than laser-treated fabrics. Therefore, the control cotton fabric tended to wrinkle more than the laser-treated fabrics. The larger values of the laser-treated cotton fabrics imply better recovery from the deformation during the test. After laser treatment, the fiber surface becomes rougher and thus the inter-fiber frictional force is increased, which will better withstand wrinkling. Therefore, laser treatment improves the wrinkle recovery of cotton fabrics. The improvement in the wrinkle recovery of cotton fabrics after laser treatment may help to reduce the conventional methods of anti-wrinkle finishing, which involve chemicals.

#### 3.1.5. Drapability

**Figure 6:**
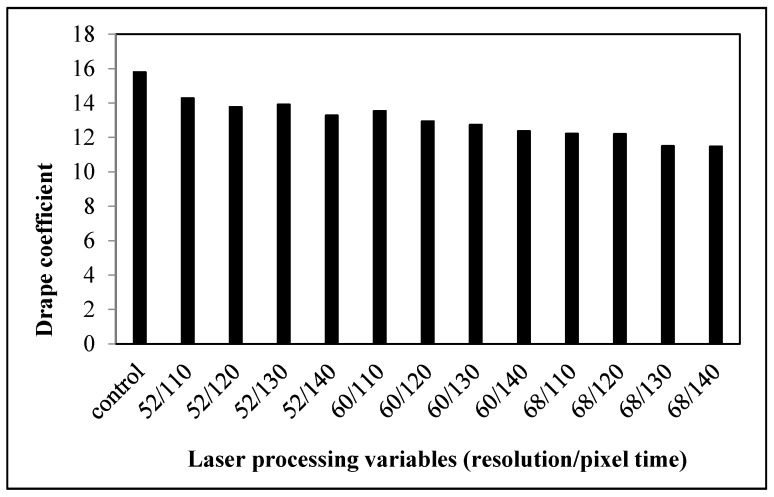
Relationship between laser processing variables and the drape coefficient of cotton fabric.

The fabric drape coefficient represents the fabric dynamic drape behavior and the fabric shape when the fabric is held at the edge. In simple terms, it is the response of the fabric towards gravity due to its own weight. The smaller the drape value, the better the draping or shape of the fabric will be. Comparison of the control fabric with laser-treated fabrics (Figure 6) shows that laser-treated fabrics have lower drape coefficients than the control fabric. This illustrates that laser treatment affects the fabric drape of cotton fabrics. Since the fiber surface is roughened after laser treatment, the inter-fiber frictional force is increased, which will better withstand draping.

### 3.2. Cotton/Polyester Blended Fabric

#### 3.2.1. Stiffness

**Figure 7:**
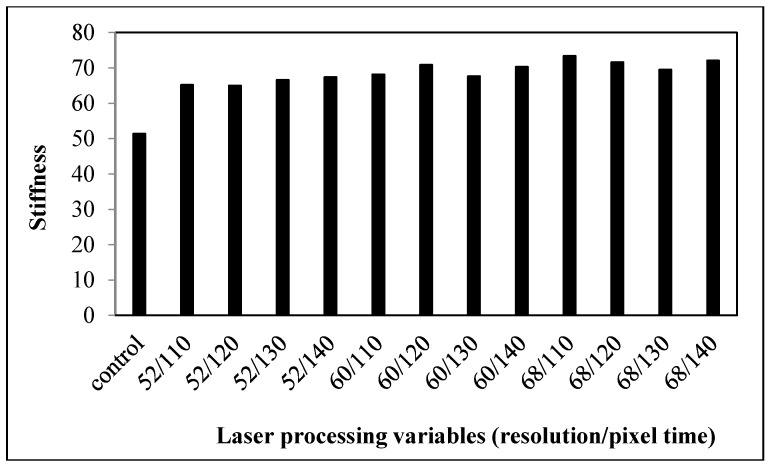
Relationship between laser processing variables and the stiffness of cotton/polyester blended fabric.

**Figure 8:**
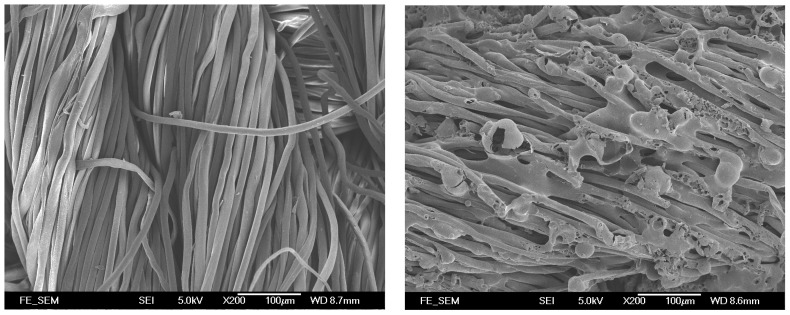
Morphological feature (200×) of control cotton/polyester blended fabric (**left**) and laser-treated cotton polyester blended fabric with variables of 52 dpi/120 µs (**right**).

Bending properties are expressed in terms of stiffness values of the control and laser-treated cotton/polyester blended fabrics, treated under different combinations of laser processing variables (Figure 7). A higher value of stiffness refers to a stiffer fabric. All the other laser-treated fabrics reveal a larger stiffness value than the control fabrics, which demonstrate that laser treatment causes an increase in stiffness of the cotton/polyester blended fabrics. In addition, the enhancement in stiffness value grows when the laser processing variables, resolution and pixel time, are increased, meaning that the fabrics become stiffer after treatment with high laser power density. In general, stiffness is affected by yarn mobility. Owing to melting and re-solidification, polyester fibers encase the neighboring cotton fibers (Figure 8) [17,18], which restricts the movement of yarns during bending. The degree of polyester melting is enlarged from a partial melting to the whole surface melting when the resolution and pixel time are increased, as shown in Table 2 [18]. As a consequence, this further intensifies the yarn restriction. As a result, the bonding of fibers and yarns together by the melted polyester enhances the stiffness of the fabrics. Bending properties have an important effect on both the handle and tailoring performance of textile materials [16].

Although too high rigidity can resist buckling and may not cause problems in garment creation generally, the comfort of wearing is lower. On the other hand, too low rigidity of the fabric gives rise to difficulties in the tailoring process. Cutting the fabrics becomes more difficult as they distort easily. In addition, it causes seam puckers more easily during the sewing operation. Therefore, with appropriate control of laser processing variables, laser treatment may resolve the problems found in some pliable fabrics during the production stage [10].

#### 3.2.2. Smoothness

**Figure 9:**
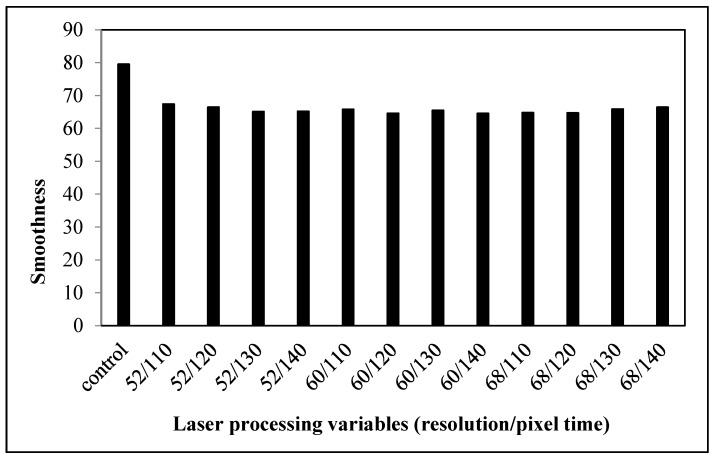
Relationship between laser processing variables and the smoothness of cotton/polyester blended fabric.

Smoothness refers to how hard one’s fingertip can push across a fabric surface. It is the surface friction and surface resistance to slipping. The smoothness of the fabrics declines after treatment with laser under different combinations of variables (Figure 9). The reduction in smoothness may be due to the formation of pores and cracks by the etching effect on the cotton fiber surface with the partial melting of polyester after laser treatment, as shown in Table 2 [10,18]. This leads to an uneven fabric surface and a relatively high surface roughness. According to the SEM images shown in our previous work [10,18] as well as Figure 8, when the laser power density is increased, more and more polyester fibers are melted. The melted polyester fibers cover the neighboring cotton fibers, in addition to the pores and cracks formed on them due to etching [10,18]. Some cotton fibers become encased under the re-solidified polyester but some may not, which leads to inconsistent surface smoothness; thus, the smoothness values are varied when the laser processing variables are changed.

#### 3.2.3. Softness

**Figure 10:**
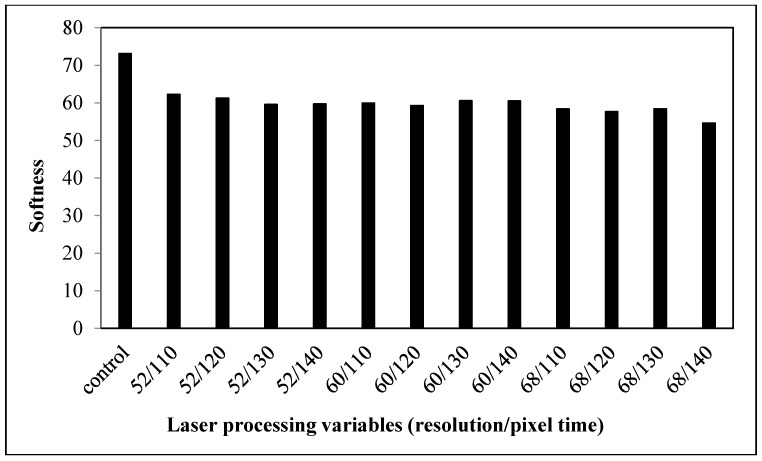
Relationship between laser processing variables and the softness of cotton/polyester blended fabric.

The smaller the softness value, the softer the fabric will be. The softness of the laser-treated cotton/polyester blended fabrics is improved compared with the control fabrics, as shown in Figure 10. As softness is related to the bulkiness of the fabrics, the increase in softness after laser treatment implies that the fullness of fabrics is enhanced [16]. Due to the melting and re-solidification of polyester fibers, the cotton fibers are encased inside the polyester fibers, thereby adding bulkiness to the fabrics (Figure 8) [18]. This would increase the fullness when fabric thickness is increased from the merging of cotton and polyester fibers.

#### 3.2.4. Wrinkle Recovery Rate

**Figure 11:**
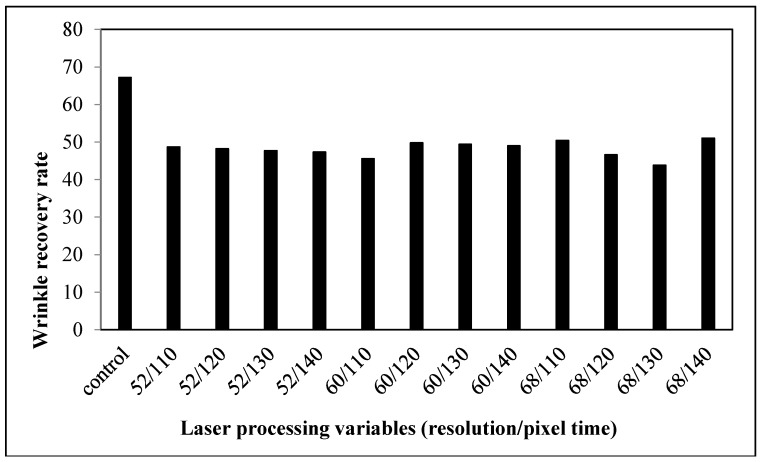
Relationship between laser processing variables and the wrinkle recovery rate of cotton/polyester blended fabric.

Wrinkles are defined as fabric deformation based on its viscoelastic properties [19]. A lower wrinkle recovery rate of laser-treated fabrics suggests that the ability to recover from folding deformation decreases when compared with the control fabrics (Figure 11). The wrinkle recovery rate is affected by the elasticity of fabrics, particularly whether it is sufficient to overcome the friction that resists the movement of the yarns and fibers. With the cotton fibers being etched and the melting of polyester fibers which encase the surrounding fibers and yarns (Figure 8), the elastic recovery power of the fabrics declines, as does its power to resist and recover from creasing.

#### 3.2.5. Drapability

**Figure 12:**
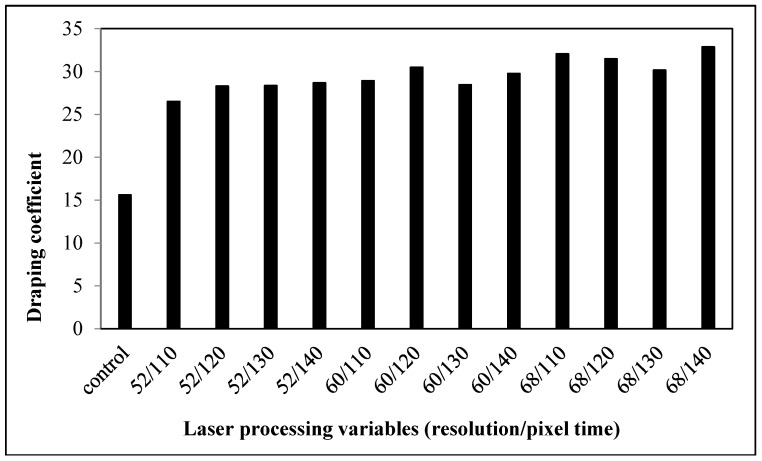
Relationship between laser processing variables and the draping coefficient of cotton/polyester blended fabric.

The draping coefficient of laser-treated fabrics is found to be larger (Figure 12), meaning that the drapability of the fabrics decreases after laser treatment. As stiffness is one of the factors affecting the draping quality, fabrics that are more flexible and are easier to drape well [20]. However, the stiffness of the laser-treated cotton/polyester blended fabrics is enhanced, indicating that these fabrics are stiffer and more difficult to drape.

## 4. Conclusions

Results obtained for the fabric hand properties demonstrate that laser treatment can be successfully used to change different aspects of the fabrics including stiffness, smoothness, softness, drapability, and wrinkle recovery. Since the aesthetic performance of a garment is governed by the quality of fabric used, the silhouette of the garment can be altered according to the change in fashion trends through the application of laser treatment on fabrics. In the case of 100% cotton woven fabrics, the fabrics were found to have a better drapability and wrinkle recovery after laser treatment. One major disadvantage of cotton as a textile fiber is its tendency to crease and wrinkle. Hence, they are treated with wrinkle-free finishing. However, these finishing treatments are usually associated with resins that release formaldehyde, which is hazardous to human health. The treatment of fabrics with laser may help reduce the use of hazardous chemicals in finishing treatments. This not only can reduce the chemicals and derivatives used, but also reduce the consumption of water and help eliminate the potential possibility of causing harmful effects to human health. As for the cotton/polyester blended fabrics, the stiffness of the fabrics was found to be relatively higher after laser irradiation. Too low rigidity of the fabric gives rise to difficulties in the tailoring process. As a result, cutting the fabrics becomes more difficult as they distort easily, and it causes seam puckers more easily during the sewing operation. Therefore, with the appropriate control of laser processing variables, laser treatment may resolve such problems in some pliable fabrics during the production stage.

## Figures and Tables

**Table 1:** Fabric specifications of two woven samples.

Fabric code	Cotton	Cotton/polyester blended fabric
Fabric Structure	3/1 Twill	3/1 Twill
Composition	100% Cotton	60% Cotton/40% Polyester *
Fabric Weight (g/m^2^)	240	229
Warp Density (end/cm)	57	48
Weft Density (pick/cm)	23	24
Warp Count (Tex)	34	29
Weft Count (Tex)	30	38
Yarn Twist	Z twist	Z twist

* Percentage was obtained by solubility test based on BS4407: 1988 with 2% tolerance.

**Table 2:** Percentage of polyester melted in the fabric.

Dpi/µs	Percentage of polyester melted in the fabric (data extracted and calculated from Reference [17])
52/110	12.26
52/120	12.45
52/130	13.26
52/140	13.61
60/110	13.61
60/120	13.78
60/130	16.76
60/140	17.86
68/110	20.44
68/120	20.53
68/130	22.29
68/140	22.73
